# Device closure of diverse layout of multi-hole secundum atrial septal defect: different techniques and long-term follow-up

**DOI:** 10.1186/s13019-019-0952-5

**Published:** 2019-07-04

**Authors:** Zeeshan Farhaj, Li Hongxin, Guo Wenbin, Wen-Long Zhang, Fei Liang, Hai-Zhou Zhang, Gui-Dao Yuan, Cheng-Wei Zou

**Affiliations:** 1 Department of Cardiovascular Surgery, Shandong Provincial Hospital Affiliated to Shandong University, No 324 Jingwu Road, Jinan, 250021 China; 2 Ultrasound Department, Shandong Provincial Hospital Affiliated to Shandong University, No 324 Jingwu Road, Jinan, 250021 China

**Keywords:** Atrial septal defect, Classification, Peratrial, Device closure, Minimally invasive surgery

## Abstract

**Background:**

There are no unanimous reports on different layouts and classifications of multi-hole secundum atrial septal defects (MHASD) and subsequent standardized occlusion techniques. The MHASD can be isolated or cribriform with variable inter-defects distance. In this retrospective study, experience-based classification and two approaches-based occlusion results are presented.

**Methods:**

We retrospectively collected and analyzed data of 150 MHASD patients from 1320 patients who underwent atrial septal defect occlusion in our institute. The MHASD patients were categorized into 4 types; type A, B, C and D and occluded under exclusive transesophageal echocardiographic guidance. According to different types, 122 patients were occluded using peratrial approach and 28 patients via percutaneous approach. In type A, single device implantation is performed to occlude the large hole and squeeze the small one. For type B single or double-device deployment was performed depending on an inter-defects distance. In type C and D, a patent foramen-ovale (PF) device was selectively positioned to the central defect to occlude the central defect and cover the peripheral ones. In peratrial approach, 8 patients underwent inter-defects septal puncture technique to achieve single-device occlusion. The intracardiac manipulation time, procedural time, double device deployment, redeployment rate, residual shunt, and proportions were analyzed between (and within peratrial technique) two techniques.

**Results:**

Successful occlusion was achieved in all 150 patients. Single device occlusion was applied in 78/84 type A and 22/37 type B patients (*p* < 0.05). Double device occlusion was more applicable to type B than A patients (*p* < 0.01). Sixteen of 21 type C and all type D patients used PF device for a satisfactory occlusion. Redeployment of the device occurred frequently in type B patients than A (*p* < 0.01). The intracardiac manipulation time and procedural time were shorter in type A than B (*p* < 0.05). The intracardiac manipulation time was also shortened in type A peratrial than type A percutaneous group (*p* < 0.05). Complete occlusion rate for all patients at discharge was 70% and rose to 82% at 1 year follow up.

**Conclusions:**

The diverse layouts and classification of MHASDs can help to choose different techniques and proper devices of different kinds to achieve better occlusion results.

## Introduction

Percutaneous device closure of secundum atrial septal defect (ASD) has been increasingly developed and applied clinically with optimal results. However, multi-hole secundum ASD (MHASD), which accounts for 10% of all ASDs [1], is still challenging in percutaneous closure especially those in big size and complex layouts. Residual shunt (RS) is common. Although previous reports [2–4] had described MHASD and the closure technique using single or double devices, no reports are published on different types of MHASDs and the corresponding way to occlude such defects. In recent years peratrial device closure of ASD has been reported effective [5, 6] which can be considered an alternative to surgical and percutaneous approaches especially in large ASD and complex MHASDs. It has the advantages of perpendicular short entry route to the interatrial septum, the high selectivity of defect-crossing, and accurate device positioning. The purpose of this retrospective study is to introduce the classification of MHASDs and to evaluate the efficacy of different methods in closing different types of MHASDs under single transesophageal echocardiographic (TEE) guidance.

## Materials and methods

### Patients’ clinical details

Between February 2003 and September 2018, a total of 150 MHASD patients underwent device occlusion in our department. Among them, 122 and 28 underwent peratrial and percutaneous device closure of MHASDs under solitary TEE guidance respectively. All patients were assessed by standard transthoracic echocardiography. Patients or guardians gave their informed written consent for the procedure. Routine examinations including electrocardiogram, chest radiograph, and blood tests were done to screen for any abnormality. The study was approved by the ethics committee of our hospital. Patients’ demographic data are presented in Tables 1 and 2.

**Table 1:** Demographic Characteristic Data of 122 Patients Using Different Peratrial Techniques

Variable	Total	Type A						Type B					Type C	Type D
		LS	MS	LS7	MS7	SS7	Total	LM	MM	LM7	MM7	Total	Cribriform	
Number of Patients	122	16	17	8	12	6	59	11	4	10	9	34	21	8
Age (years)	20 ± 20 (0.4–64)	25	3	32	3	3	16 ± 19	31	45	25	31	30 ± 18	3.4 (0.7–61)	15 (3–58)
Gender (F/M)	83/39	12/4	7/10	6/2	6/6	2/4	33/26	9/2	3/1	8/2	9/0	29/5	18/3	5/3
Weight (kg)	37 ± 22.8 (6–85)	54	14	59	15	13	32 ± 23	58	61	55	62	52 ± 15	15	31
Maximum ASD size (mm)	12.7 ± 6.9 (2.8–28)	20	9	19	7	4	13 ± 7	19	10	19	11	16 ± 5	5 (3–15)	9
Median device Size (mm)	22 ± 7 (6–36)	27	16	26	14	1818	22	30	22	24	18	24	PF2525	PF2525
D Value	5.3 ± 3.3 (0–16)	5	3	7	3	4	5 ± 3	6	8	5	7	7 ± 4	–	–
Double-device (n)	22	0	0	2	4	0	6*	4†	0†	7†	4†	15*	1	0
PF-Device (n)	39	0	2	0	4	5	11	0	1	1	2	4	16	8
Redeployment (n)	43	4	0	3	3	1	11¶	6	3	7	4	20¶	8	4
ICMT (min)	17 ± 17.5 (2–75)	6	4	13	11	11	13 ± 16§	8	29	24	26	26 ± 22§	20	12
Procedural Time (min)	68.8 ± 26 (30–175)	54	47	65	56	57	60 ± 19‡	72	90	86	90	89 ± 31‡	58	55

*LS* Large-Small, *MS* Moderate-Small, *SS* Small-Small, *LM* Large-Moderate, *MM* Moderate-Moderate; The numeric “7” denotes the inter-defects distance between the two relatively larger holes of ≥7 mm.; *ASD* Atrial septal defect; *D value* Difference between device and defect size, *PF* PF devices, *ICMT* Intracardiac manipulation time; *p = < 0.05; †*p* = < 0.05; ¶*p* = < 0.05; §*p* = 0.05; ‡ *p* < 0.05

**Table 2:** Demographic Data of Percutaneous Patients

Variable	Total	LS	MS	SS	MS7	LM
Patients (n)	28	4	8	9	4	3
Age (years)	26.55 ± 22.5 (1–63)	25 ± 17	41.8 ± 13.4	3.9 ± 1.6	24.4 ± 26.6	43 ± 9.6
Gender (F/M)	23/5	3/1	6/2	7/2	4/0	3/0
Weight (kg)	41.8 ± 22.3 (11–76)	48 ± 26	54.8 ± 16.6	16.3 ± 3.3	42 ± 18.7	66 ± 2.5
Maximum ASD size (mm)	1.1 ± 6.25 (3–23)	19 ± 3.5	11.5 ± 3.41	3.5 ± 0.6	12.3 ± 1.3	19 ± 4.2
Device Size (mm)	20.9 ± 6 (6–35)	25 ± 5	22.8 ± 6	PF1818	17.5 ± 3	28 ± 2
Ordinary Device (n)	15	4	5	1	2	3
PF Device (n)	13	0	3	8	2	0
Redeployment (n)	6	1	2	1	1	1
ICMT (min)	20 ± 11 (3–50)	23.3 ± 13.8	27 ± 13.5	20.7 ± 8.9	8.7 ± 6	19 ± 9.5
Procedural Time (min)	29.7 ± 17.7 (10–75)	28.6 ± 17.6	33 ± 20.6	29 ± 17	37.7 ± 23.9	21 ± 9
Complete Occlusion-rate 3 month (%)	92.6%	100%	87.7%	100%	75%	100%

*L* Large, *S* Small, *M* moderate, 7 = denotes inter-defects distance, *ICMT* Intracardiac-manipulation-time

#### Inclusion criteria

(1) presence of ≥2 holes of secundum ASD; (2) age of 6 months or older; (3) signs of right ventricular overload; (4) minimal shunt in the presence of symptoms (arrhythmias (paradoxical emboli), transient ischemic attacks, etc.); (5) presence of a rim of ≥4 mm from the implanting hole of the MHASD to the superior or inferior vena cava, coronary sinus, mitral valve, and right upper pulmonary vein.

#### Exclusion criteria

(1) coexisting cardiac problems requiring surgical correction; (2) one of the defects size of > 35 mm in diameter; (3) contraindication for antiplatelet therapy.

#### Classification of MHASD

The following classification was used to decide about closure techniques for all the patients. According to the ASD size, constituents, morphological layout and the inter-defects distance (IDD) the MHASDs were classified into four types as Type A, B, C and D. The defects were sized as large (> 15 mm), moderate (≥5 - ≤15 mm) and small (< 5 mm). The IDD was defined as the distance from the central defect (implanting hole) to the surrounding hole which needs to be covered. The MHASDs were seen as “central defect” formation. The central defect was the occlusion point. This means a defect was usually in the center of other defects. Two central defects formation was frequently encountered in type B and type C. The central defect could be large, moderate or small. This classification can be explained as follows for all types;

In Type A there is one big defect (either large, moderate or small) associated with one or more small defects. The central defect is a big defect among all. The type A is sub-grouped as large-small, moderate-small, and small-small with the furthest IDD of < 7 mm, and large-small-7, moderate-small-7 mm, and small-small-7 mm, with the furthest IDD of ≥7 mm.

In Type B, there are two big defects (large or moderate). Each of the big defects may be associated with one or more small holes. The central defect is one of the big defects or both. The type B is sub-grouped as large-moderate, moderate-moderate, with the IDD between the two big holes of < 7 mm, and large-moderate-7 mm and moderate-moderate-7 mm, with the IDD between the two big holes of ≥7 mm.

Type C was seen as the cribriform where multiple small defects or including one moderate hole with the number ≥ 5 would form this formation. The central defect is a small defect in the middle of the formation.

**Fig. 1:**
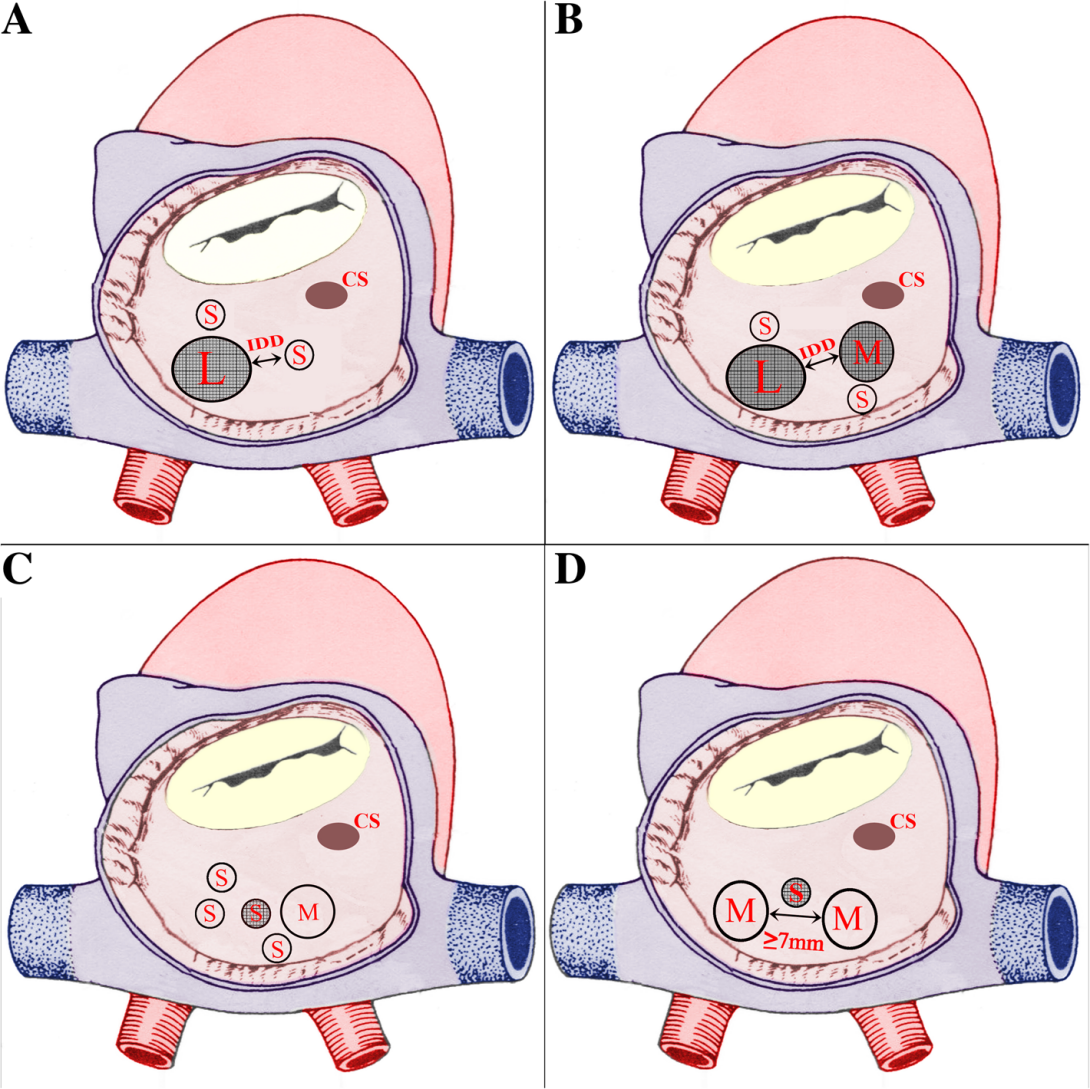
Classification of multi-hole atrial septal defects. **a** Type A, Large-Small hole configuration accompanying small defect with different IDD. **b** Type B, Large-Moderate configuration accompanying small defects with different IDD. **c** Type C, cribriform formation, with the defect number of ≥5. **d** Type D, Moderate-Small-Moderate morphology, IDD ≥7 mm. (L = Large; S = Small; M = Moderate; IDD = Inter-defects Distance; Shadow = Central implanting defect; CS = coronary sinus)

Type D was seen as a small central defect with one moderate defects on both sides (Fig. 1). This classification along with IDD would influence on the device selection.

#### Device and delivery system

**Fig. 2:**
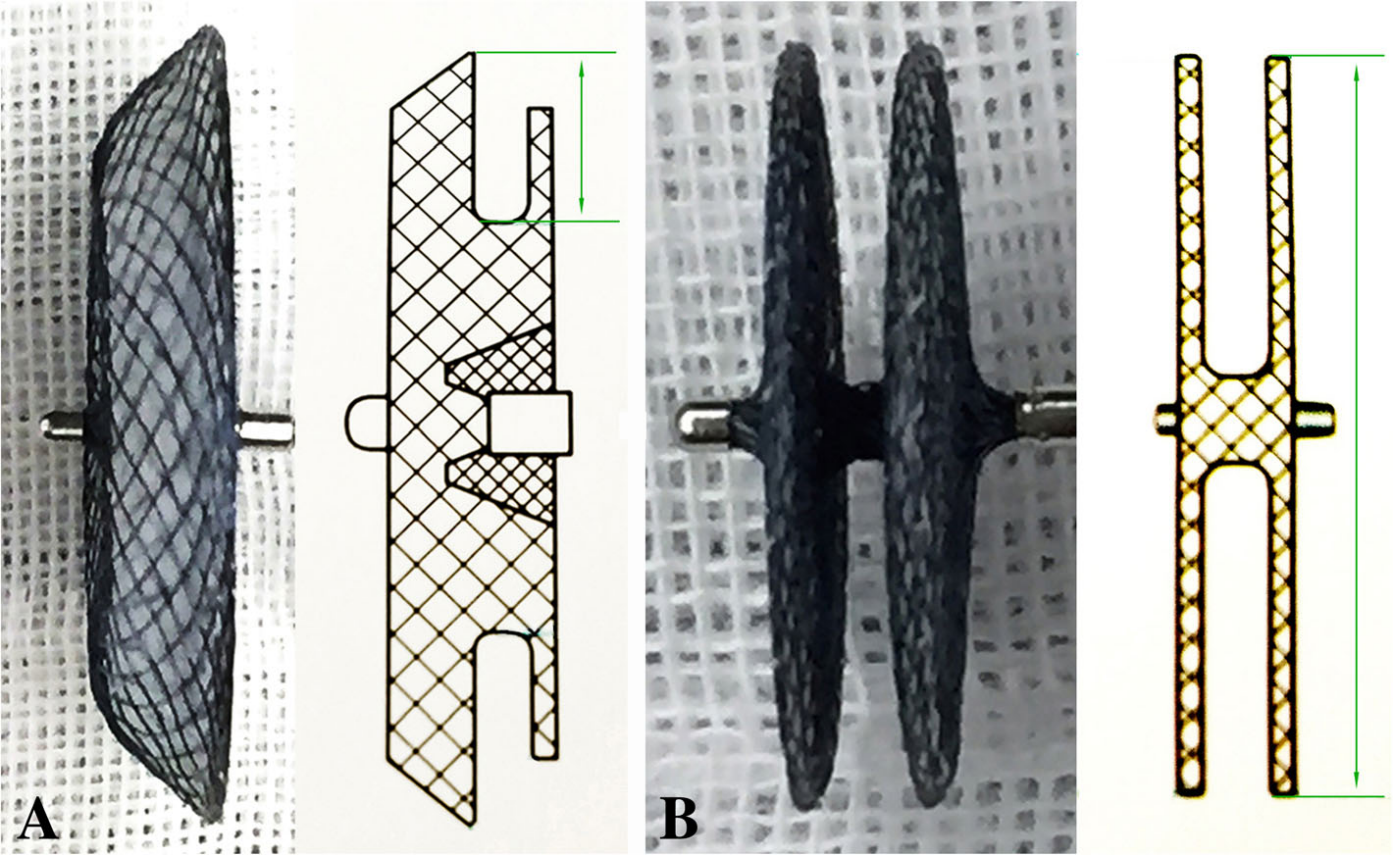
Occlusion devices. **a** ordinary atrial septal occluder, with the left disk 6, 7 or 8 mm larger than the connecting waist for device sizes of ≤10, > 10–30, and > 30 mm respectively. **b** PF device, distinct from ordinary one by the smaller waist and identical diameter of wide-edged left and right disks

Two types of devices (Starway Medical Technology, Inc., Beijing, China) were used in this study. The ordinary atrial septal occluder and patent foramen-ovale (PF) device, based on Amplatzer septal occluder and multifenestrated septal occluder respectively. The diameters of right and left disk, which are PF1818, PF2525, PF3030, and PF3535 mm, determine the size of the PF device (Fig. 2).

The delivery system (Starway Medical Technology, Inc., Beijing, China) includes a long and short plastic sheath with a sidearm and a delivery cable, which is available in size of 7F to 14F [5–8]. The dilating sheath was used as a guiding sheath to cross the defect selectively in some patients.

### Procedure and device selection

Patients were placed in a supine position. Under general anesthesia, a complete preprocedural TEE (Philips IE33; Philips Healthcare, Best, Netherlands) was performed to assess all the defects’ size, morphological layouts, IDD, surrounding structures and rims as part of closure protocol. Peratrial technique has been reported previously [5, 6]. In summary, a 1.5 to 2 cm incision was made in right 4th parasternal intercostal space. The pericardium was incised and suspended. Two purse-string sutures were placed on the right atrium with polypropylene 4–0. A puncture was made in the purse-string. The delivery sheath was advanced through the puncture under the TEE guidance and an appropriately sized device or two were deployed. The inter-defect septal puncture (IDSP) technique was chosen in some patients. The IDSP involved a puncture needle, advanced through purse-string. The needle was pointed to the septum in the middle of two defects. Under the TEE guidance, the needle was advanced to the left atrium through the inter-defects septum. A guidewire was passed through puncture needle to left atrium then the rest of the delivery system followed.

**Fig. 3:**
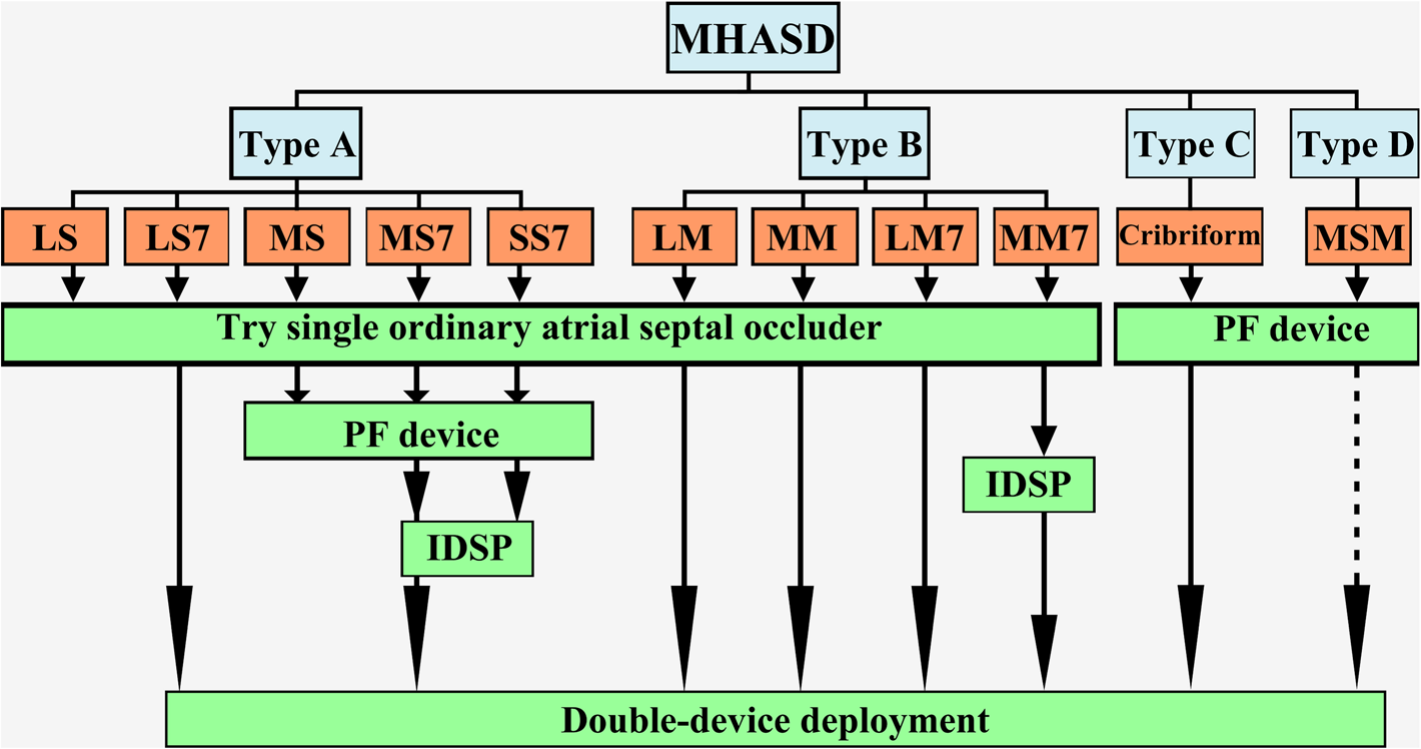
Device selection flow chart. Device selection flow chart. (LS = large-small; MS = moderate-small; SS = small-small; LM = large-moderate; MM = moderate-moderate; MSM = moderate-small-moderate hole configuration, IDSP = inter-defects septal puncture. The numeric “7” denotes inter-defects distance of ≥7 mm.) Dotted arrowhead line denotes possibility of double device deployment

The device selection process is shown in Fig. 3. In type A, the ordinary device size was chosen with the size 4 to 6 mm larger than the maximum diameter of the large defect. The delivery sheath was advanced through the large defect to the left atrium. The device was deployed to occlude the large defect and squeeze the surrounding small ones. The unsatisfactory device would be replaced with a proper smaller or bigger one. In moderate-small-7 mm, small-small-7 mm, and moderate-small subtypes, a PF device might be chosen for closure for the first time or after trying the single ordinary device, with the size twice the sum of IDD and the diameter of the larger non-implanting hole which need to be covered. The small defect was thought to be the central defect rather than the big one, once a PF device was used.

In type B, most large-moderate, moderate-moderate and some of the large-moderate-7 mm and moderate-moderate-7 mm subtypes, especially those where the interatrial septum was of an aneurysmal type, or the inter-defects septum was soft, an ordinary single device was implanted in the large defect. The device size was selected by adding 6 to 9 mm to the maximum diameter of the large defect in order to occlude the large and squeeze the small MHASDs.

**Fig. 4:**
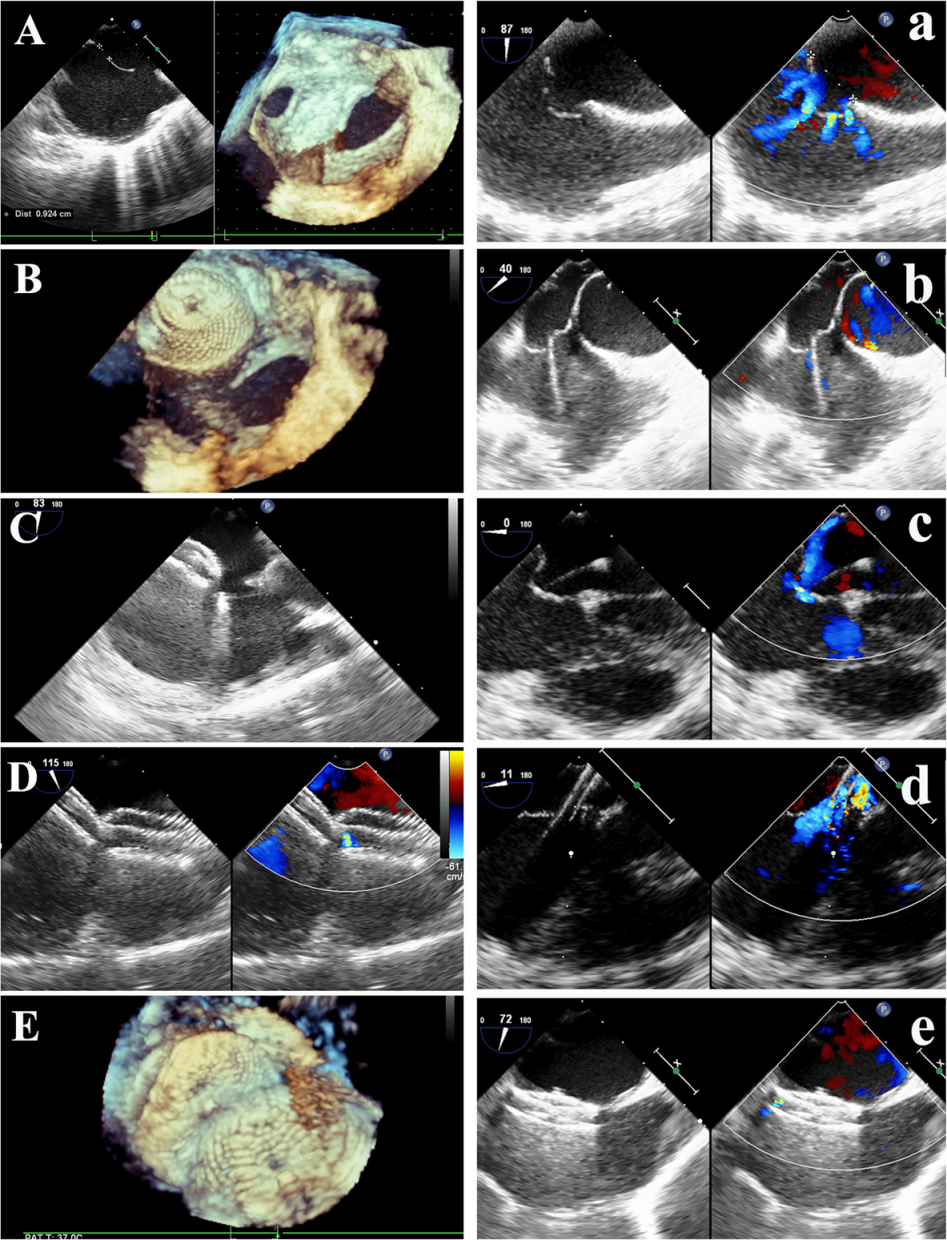
Device closure of type B and C multi-hole atrial septal defects. **A** Defects layout of type B. **B** The small defect was occluded first. **C** Then the delivery sheath was advanced through the large defect. **D** and **E** Double-device occlusion was achieved. **a** Defects configuration of type C cribriform. **b** The guiding sheath was advanced through the middle small central defect **c** introducing the flexible guidewire through the defect. **d** The delivery sheath was passed through the defect over the wire. **e** The single device occluded all the defects

In type A large-small-7 mm, moderate-small-7 mm subtypes and type B, especially large-moderate-7 mm and moderate-moderate-7 mm subtypes, if single device deployment was not feasible, double-device deployment was performed. Both devices were connected with a device-stay suture before retracting into the sheath [7]. The inferior small central defect was usually occluded first with the device 2 to 4 mm larger than the maximum diameter of the defect. After positioning and testing with a push-pull maneuver, the small device was released. Then a big device was selected and deployed in the large central defect with the size 3 to 7 mm larger than the hole (Table 3) (Fig. 4A-E).

**Table 3:** Clinical Data of Simultaneous Double-Device Occlusion in 22 Patients

Patient number	Age (y)	Diameter of big CD (mm)	Device size of big CD (mm)	D value 1 (mm)	Diameter of small CD (mm)	Device size of small CD (mm)	D value 2 (mm)	Inter defects distance (mm)	Order of device closure	Types	RS
1	5	18	24	6	8	12	4	16	SB	LM	N
2	39	15	22	7	13	20	7	12	SB	LM	N
3	2	7	8	1	4	6	2	8	SB	Type C	N
4	30	11	18	7	6	12	6	14	BS	MM	N
5	2	7	10	3	3	6	3	14	SB	MS	N
6	13	10	10	0	5	8	3	20	BS	MM	N
7	8	20	24	4	12	16	4	9	SB	LM	N
8	3	13	16	3	3	6	3	9	SB	MS	N
9	4	10	12	2	5	8	3	11	SB	MS	**Y**
10	3	6	8	2	3	4	1	12	SB	MS	N
11	10	14	18	4	7	10	3	19	SB	MM	N
12	8	23	26	3	3	6	3	9	SB	LS	**Y**
13	36	9	14	5	6	8	2	12	BS	MM	N
14	49	21	28	7	9	12	3	16	SB	LM	**Y**
15	10	16	20	4	4	6	2	11	SB	LM	N
16	10	19	22	3	4	6	2	7	SB	LS	N
17	34	18	22	4	8	12	4	10	SB	LM	N
18	13	23	26	3	13	16	3	3	SB	LM	N
19	58	16	18	2	7	12	5	3	SB	LM	**Y**
20	56	28	36	8	8	PF3030	–	19	BS	LM	N
21	48	19	26	7	12	PF2525	–	3	BS	LM	N
22	64	16	22	6	13	16	3	2	SB	LM	N
Mean ± SD	20 ± 19	15 ± 6	19 ± 7	4 ± 2	7 ± 3	10 ± 4	3 ± 1	11 ± 5	–	–	–

*CD* central defect, *D value* Difference between device and defect size, *SB* small-hole device closure first then the big hole, *BS* big-hole device closure first then the small hole, *RS* Residual shunt at 3 months follow-up, *LM* Large-Moderate, *MS* Moderate-Small, *MM* Moderate-Moderate

**Fig. 5:**
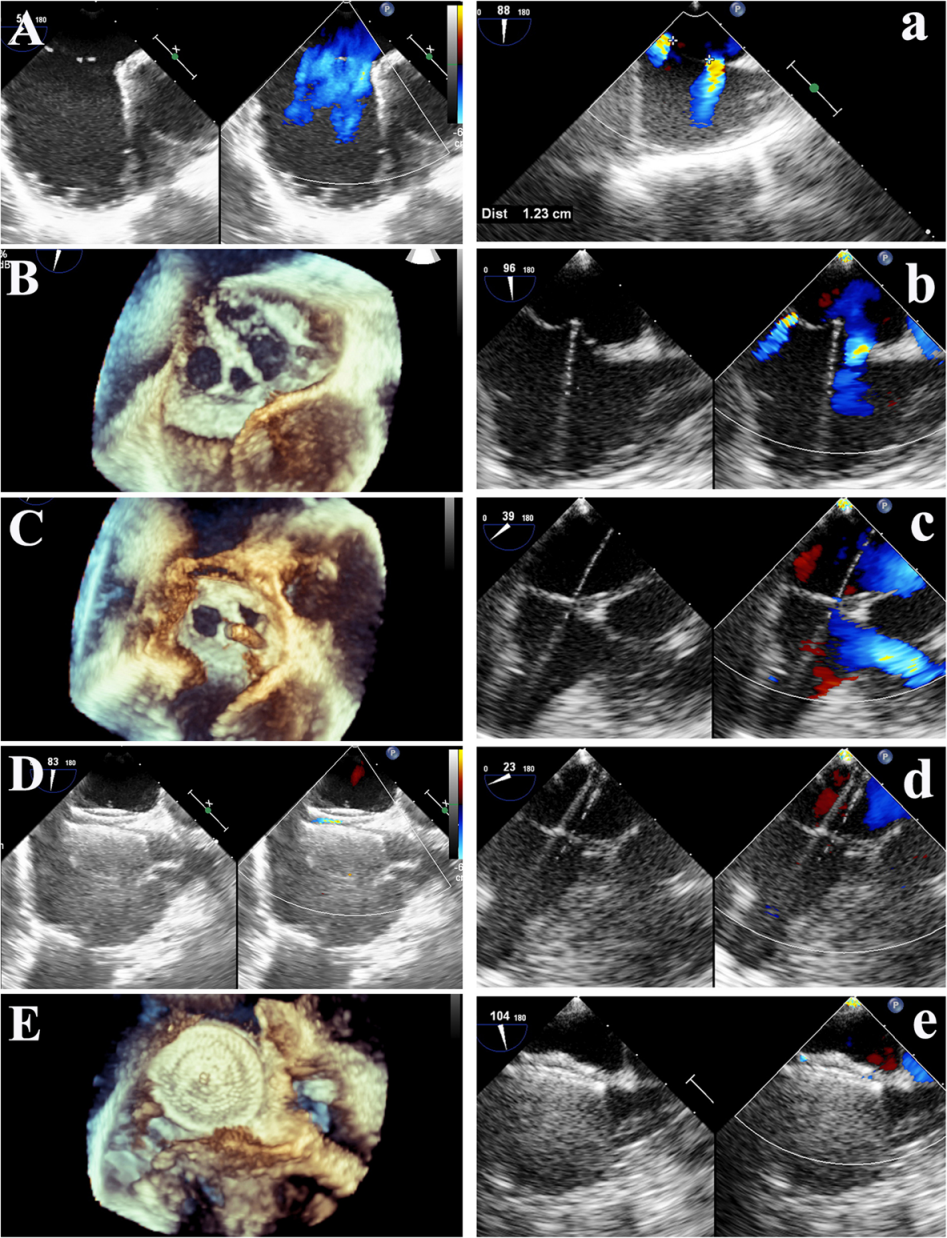
Device occlusion of type D multi-hole atrial septal defects and inter-defects septal puncture technique. **A** and **B** Type D Moderate-small-moderate layout. **C** The delivery sheath was crossing the middle small hole. **D** and **E** All defects were covered by a single device. **a** Defects layout. **b** The puncture needle was progressed through the chosen optimal midpoint. **c** A flexible guidewire and **d** the delivery sheath were passed through the puncture point into the left atrium. **e** Both defects were closed by a single device

For the type C cribriform (Fig. 4a-e), the PF device was selected with the size twice the distance between the middle implanting hole and the farthest non-implanting hole. For type D (Fig. 5A-E), the PF device was chosen by the sum of the maximum diameter of the larger defect and the farthest IDD. In type D if the sum of diameter of the defects and the IDD was larger than that of PF3535 diameter then the double device deployment might be applied.

In type A moderate-small-7 mm, small-small-7 mm, and type B moderate-moderate-7 mm, if RS was still significant after the above maneuver, an IDSP technique was performed (Fig. 5a-e). A PF device was selected, with the size equal to the sum of the maximum diameter of the larger hole and half of the width of IDD, and deployed. In the IDSP technique, an iatrogenic small central defect is created.

Sometimes the guiding sheath was needed to introduce a flexible guidewire to cross the central small implanting defect selectively. Then the sheath was passed through the defect for device deployment.

After device positioning, the device configuration, the RS, and the presence of interference with the adjacent structures were assessed. Then the occluder was released and the device-stay sutures were removed. Patients with the RS of more than 3 mm in size, after single device maneuver, were considered for other options as PF device, IDSP or double-device implantation.

The sizes of the defects, intracardiac manipulation time (ICMT), total procedural time and RS were recorded.

#### Follow-up

All patients were given prophylactic antibiotics and discharged 3rd to 5th postoperative day. Aspirin (3-4 mg/kg/day) was continued for 6 months. Follow-up electrocardiogram and transthoracic echocardiography were scheduled at discharge, 1, 3, 6, 12 months and yearly thereafter. Patients were considered to have successful ASD closure if they had no or RS < 3 mm as assessed by TEE.

#### Statistical analysis

Data are expressed as median or mean ± standard deviation and range. The ICMT, procedural time, maximum ASD size and device size, between the two groups were compared with the independent samples t-test. Statistical comparisons of proportions were analyzed using a chi-square or Fisher’s exact test (Stata10.0; StataCorp LP, College Station, TX). A probability value of less than 0.05 was defined as statistical significance.

## Results

Successful occlusion was achieved in all 150 patients with MHASD including 28 patients underwent percutaneous closure. Single and double-device deployment was done in 100 and 22 patients, respectively in peratrial approach patients. The IDSP technique was applied in 8 patients. All percutaneous approach patients were in type A, except 3 patients with a type B MHASD which had a soft interatrial septum and a short IDD. (Tables 1 and 2).

Single occluder was more commonly used in type A patients (78/84) compared with type B (22/37, *p* < 0.05). Single PF device was frequently used in type A-moderate-small-7 mm (6/16), small-small-7 mm (5/6) SS (8/9) subtypes, type C (16/21) and D (8/8) patients. Four earlier patients with type A moderate-small-7 mm received ordinary double devices, while 5 patients with type C-cribriform underwent 1 double device and 4 single ordinary device deployment due to PF device stock out. The IDSP technique was applied to 3 moderate-small, 3 moderate-moderate, and 2 small-small-7 m. All of them had no RS at 3 months follow-up.

Peratrial double-device deployment was performed in 22 patients and was more frequently used in type B (15/34) than type A (6/59) (*p* < 0.01). In Type B the subtypes with IDD ≥7 were more likely to undergo double or PF device deployment as compared to those with IDD < 7 (*p* = 0.01). The small implanting hole which was located inferiorly in 15 patients, was occluded first in 16 patients and the big hole first in 6 patients, which was located inferiorly in 4 patients (Table 3). The ICMT and procedural time were longer in type B than type A (both *p* < 0.01).

Redeployment of the device occurred in 43/122 patients (35.2%). The device was replaced with a larger size in 33/43 subjects due to instability or RS. In 10/43 subjects, the device was replaced with a smaller size owing to infringement, overestimation or making room for double-device deployment. Redeployment of the device occurred frequently in type B patients (*p* < 0.01) (Table 1).

All patients were followed up for a period of 3 months to 15 years (median, 48 months) with transthoracic echocardiography. At 3 months follow-up, the RS occurrence rates were 18, 20.6, 40, 12.5 and 13.6% in type A, B, C, D, and double-device deployment, respectively. The total RS rate was 22.3%. The residual shunt occurrence was more in single ordinary device occlusion (8/45) patients as compared to those with PF device or double device deployment (3/33) (*p* > 0.05).

The maximum ASD size was larger in type A peratrial than the percutaneous patient (*p* > 0.05). Similarly, the devices used in type A peratrial occlusion were larger than the percutaneous group (*p* > 0.05). The ICMT in type A peratrial group was significantly shorter than the percutaneous group (*p* < 0.05). The procedural time, on the other hand, was shorter in the percutaneous group as compared to type A peratrial (*p* < 0.05).

Complete occlusion rate at discharge for all (150) patients was 70% and rose to 82% at 12 months follow up (Table 4). All RS at 12 months follow up were < 3 mm in diameter. During the follow-up period, there was no device dislocation, thrombosis, hemolysis, or infective endocarditis detected in either type of MHASDs. The patients in both groups were discharged at 3rd-5th day postoperatively. In the peratrial group, no patient experienced pericardial effusion or postpericardiotomy syndrome. The peratrial device closure patients experienced minimal postoperative pain. There were no other early or late complications of surgery.

**Table 4:** Residual Shunt Follow-up After Device Closure of Different Types of Multi-Hole Atrial Septal Defects

Types		IADR (*n* = 150)	Discharge (*n* = 150)	1 m (*n* = 150)	3 m (*n* = 150)	6 m (*n* = 136)	1 y (*n* = 118)	2 y (*n* = 104)	3 y (*n* = 81)	> 5 y (*n* = 43)
Type A (*n* = 84)	LS (*n* = 20)	10	9	6	4	4	3	3	3	2
	MS (*n* = 25)	5	3	3	3	3	3	2	1	1
	SS (*n* = 9)	0	0	0	0	0	0	0		
	LS7 (*n* = 8(	5	5	5	5 ^a^	4	4	3	2	
	MS7 (*n* = 16)	8	5	4	4 ^a^	4	2	1	1	
	SS7 (*n* = 6)	1	1	0	0	0	0	0	0	0
Type B (*n* = 37)	LM (*n* = 14)	4	3	3	3 ^a^	3	3	2	1	1
	MM (*n* = 4)	1	1	1	1	1	1	1		
	LM7 (*n* = 10)	6	3	2	2 ^a^	2	1	1		
	MM7 (*n* = 9)	3	3	2	2	2	1	1	1	
Type C (*n* = 21)	Cribriform (*n* = 21)	12	11	9	8	7	6	4	2	1
Type D	(*n* = 8)	3	1	1	1	1	0	0	0	0

*LS* Large-Small, *MS* Moderate-Small, *LS7* Large-Small with inter-defects distance 7 mm, *MS7* Moderate-Small with inter-defects distance 7 mm, *LM* Large-Moderate, *MM* Moderate-Moderate, *LM7* Large-Moderate with inter-defects distance 7 mm, *MM7* Moderate-Moderate with inter-defects distance 7 mm, *SS* Small-Small, *IADR* immediately after device release, ^a^One of the patients with double devices or a PF device had residual shunt

## Discussion

Since 2001 1320 patients have undergone peratrial or percutaneous device closure of ASDs under TEE guidance in our center. The incidence of MHASDs is 11% which correlates to that reported by Podnar et al. [1].

### Clinical implications for the classification of MHASDs

One principal rule, we must obey, is that the MHASDs are better to be occluded using a single device.

In type A we opted to occlude the defects with a single device. Except for ordinary devices, PF device is applicable in moderate-small, small-small, moderate-small-7 mm and small-small-7 mm subtypes. If the preoperative assessment was that the defects could be occluded using a single device without RS, the percutaneous approach was chosen.

In type B single device occlusion was tried first then double devices. Although more double devices were used in type B large-moderate-7 mm and moderate-moderate-7 mm versus large-moderate and moderate-moderate, yet we think that the latter two still hold the possibility of double device deployment. Figure 3 does not mean that every patient should be tried a single ordinary device first. It’s just an idea of device selection order. If the interatrial septum was thick, or the IDD was wide enough, double-device or a PF device could be selected directly. If the inter-defects septum was wide (with IDD > 7 mm) but soft or aneurysmal, we would occlude the big central defect by the larger device and moderate or small defects were squeezed by the waist to achieve single device occlusion. In type B, percutaneous closure was rarely indicated unless the inter-defects septum was soft or aneurysmal, the occlusion was achieved following the above method.

In type C and D, a single PF device was deployed in the central small implanting hole. Many cribriform MHASDs have aneurysmal interatrial septum. The selective crossing of the central small defect is challenging. Sometimes the fine and the sharp-tipped dilating sheath is used to guide the wire to cross the defect. The PF device can also strengthen and flatten the aneurysmal interatrial septum.

### Double-device deployment and conversion between types

In double-device closure, we usually occlude the inferior, or small central defect first then the big central defect. Because most small ASDs were located in the inferior part of the septum, it would be difficult to occlude if the superior larger central defect was occluded first. The small-hole device usually had greater anchor and stability. In this way, the big-hole device can “hug” the device to decrease the exposed device-area. Whereas, if the large hole was located inferiorly, it was occluded first. The superior small hole can be occluded with a smaller device due to the rigid support from the lower large device. This might decrease the risk of aortic erosion of the small device.

In some occurrences where different types are present in the same individual, we tend to classify them to the closest type. For example, we defined type C to be the formation of multiple small holes or one moderate hole combined with multiple small holes. If two moderate holes were in the cribriform formation, it converts into a type B moderate-moderate. In type C, if the moderate hole is more than 10 mm in diameter, the interatrial septum is wide and soft, or the big PF device is not available, we can convert this type C to type A using an ordinary device to occlude the big and squeeze the small holes. Another example, if IDD is too wide in type D, even the biggest PF device could not completely cover the peripheral holes. We can convert this type D formation to type B moderate-moderate.

### Advantages of peratrial device closure of MHASDs

The Peratrial device closure of ASD is very common in Asia (China), and other places [5, 6, 9–12] due to multiple advantages as following. A detailed discussion comparing peratrial device closure to traditional surgical repair and transcatheter occlusion has been done previously [5].

1. Peratrial closure is more accurate than percutaneous closure due to perpendicular short entry route and easiest maneuverability of a short delivery sheath. The sheath permits easier defect crossing and more accurate device positioning, especially those with an aneurysmal interatrial septum. Theoretically, the peratrial technique can decrease the possibility of RS.
2. The short rigid delivery sheath or the guiding sheath has high selectivity in crossing the small defect, especially in type C and D.
3. Double-device implantation is more feasible in peratrial approach. The device-stay suture improved safety by preventing device embolization. Double-device implantation did not increase the likelihood of thrombus formation and conduction abnormality in this study.
4. IDSP technique is impractical in the percutaneous approach. The difficulties would come from not only the puncture itself but also the soft, aneurysmal interatrial septum.
5. No radiation exposure
6. Cheaper than the percutaneous approach in third world countries [5, 13].

### Inter-defects septal puncture technique

The idea of this technique originated from type D. It decreased the possibility of using double devices to occlude the MHASDs. This technique can only be used in type A moderate-small-7 mm, small-small-7 m, and type B moderate-moderate-7 mm, with the IDD of more than 7 mm because the largest available PF device is PF3535. Care must be taken not to cover the coronary sinus or impinge on the mitral valve when using a big PF device. Previously atrial remodeling to deploy the single device in MHASDs has been reported [9, 14]. The IDSP technique was developed thinking of drawbacks of the atrial remodeling; rim support might be lost, single defect becoming larger, device instability and embolization. In some cases with an aneurysmal type of interatrial septum and small left atrium, the IDSP is not recommended. It increases the risk of left atrial posterior wall injury.

### Residual shunt

In type A and B, the combinations of large-small, large-small-7 mm, large-moderate and large-moderate-7 mm were more prone to RS. The more the size of MHASDs (the first two big) approached similarity the easier the RS occurred in patients using a single device. RSs also frequently occurred in type C cribriform patients. We think RS of less than 3 mm is hemodynamically insignificant. The patients will benefit little by double-device implantation which could increase the likelihood of thromboembolism and arrhythmia. In our series, most of the RSs disappeared gradually during the follow-up period. Our results for double-device closure were concordant with the previous reports [2–4]. Yang et al. [2] reported an 88.6% complete occlusion rate for double device patients at 6 months follow up. Awad et al. [3], reported 100% occlusion at 6 months follow-up in 33 patients, where the double device was used in 32 patients. Mahadevan et al. [4] also reported similar results to our double device occlusion patients where 36 patients underwent occlusion and complete occlusion rate 12 months was 88%. As to different types of MHASDs, the RS rate is highest in type B patients even with double devices.

### Study limitations

In some types and subtypes of MHASDs, there are few patients. We have only 8 patients who underwent IDSP technique. Less experience is available. In our series, we have not met MSM patient with very wide IDD in which even the biggest PF 3535 can’t cover all the holes and double-device deployment might be chosen. Only 22 patients have been implanted double devices which might incur more complications. Further studies are required to establish long-term results in a larger patient population. General anesthesia is still one of the shortcomings in peratrial technique. This classification is not strict enough for a technique or device selection.

## Conclusions

According to diverse layouts of MHASDs, proper selection of different kinds of devices and different techniques can achieve better occlusion results. Type A MHASD with a short IDD is more suitable for a percutaneous approach. In type A, single device implantation is performed to occlude the big hole and squeeze the small one. In type B, to try single device closure first then double-device implantation. In type C and D, the PF device is selected to occlude the middle hole and cover the peripheral holes. Some of the type A and B, MHASDs can be turned into a type D using IDSP technique to achieve a single-device occlusion.

## Data Availability

The datasets generated during and/or analyzed during the current study are not publicly available due to privacy but are available from the corresponding author on reasonable request.

## Abbreviations

ASD: Atrial septal defect
ICMT: Intracardiac manipulation time
IDD: Inter-defects distance
IDSP: Inter-defects septal puncture
MHASD: Multi-hole secundum atrial septal defect
RS: Residual shunt
TEE: Transesophageal echocardiography
